# A Model of How Shifting Intelligence Drives Social Movements

**DOI:** 10.3390/jintelligence9040062

**Published:** 2021-12-13

**Authors:** Noah F. G. Evers, Patricia M. Greenfield

**Affiliations:** 1 Department of Psychology, Harvard College, Cambridge, MA 02138, USA; noahevers@college.harvard.edu; 2 Department of Psychology, University of California, Los Angeles, CA 90095, USA; 3 Department of Human Evolutionary Biology, Harvard University, Cambridge, MA 02138, USA

**Keywords:** intelligence, social movements, theory of social change, cultural evolution, and human development, social intelligence, practical intelligence, abstract intelligence, COVID-19, cultural evolution, adaptive intelligence, George Floyd protests

## Abstract

Based on the theory of social change, cultural evolution, and human development, we propose a mechanism whereby increased danger in society causes predictable shifts in valued forms of intelligence: 1. Practical intelligence rises in value relative to abstract intelligence; and 2. social intelligence shifts from measuring how well individuals can negotiate the social world to achieve their personal aims to measuring how well they can do so to achieve group aims. We document these shifts during the COVID-19 pandemic and argue that they led to an increase in the size and strength of social movements.

## 1. Introduction

Based on the theory of social change, cultural evolution, and human development (Evers et al. 2021; Greenfield 2009, 2016, 2018; Greenfield et al. 2021), we propose a mechanism whereby increased danger in society (as indicated by mortality rate and resource scarcity), plus smaller social groupings, trigger evolutionarily conditioned shifts toward the forms of intelligence valued in the small-scale subsistence ecologies omnipresent in earlier human history. Under these conditions, there are two shifts in valued forms of intelligence: 1. *practical intelligence* rises in value relative to *abstract intelligence*; and 2. social intelligence shifts from measuring how well individuals can negotiate the social world to achieve their personal aims to how well they can negotiate the social world to achieve group aims. We document these shifts during the pandemic as society became more dangerous and social units became smaller. We then argue that these shifts in the valued forms of intelligence have led to an increase in the size and strength of social movements. Lastly, we conclude that, on average, throughout human history, these psychological responses likely contributed to humankind’s success by creating the most adaptive society for each set of changing environmental conditions. However, since humans seem to employ adaptive intelligence at the community level, one community furthering its aims can cause harm to another community.

## 2. Theory of Social Change, Cultural Evolution, and Human Development

The theory of social change, cultural evolution, and human development is a predictive model of how changing sociodemographic variables shift psychological and behavioral variables consistent with archetypal ecological variables (Evers et al. 2021; Greenfield 2009, 2016, 2018; Greenfield et al. 2021). Sociodemographic variables, all of which induce value changes, include formal education, urbanization, and communication technologies (e.g., Manago 2012; Weinstock et al. 2014). The relevant sociodemographic variables in the case of COVID are mortality rate, resource scarcity, and community size. The two ecological archetypes are subsistence and commercial ecologies. The former is associated with a collectivistic value system, and the latter with an individualistic value system. Rather than affecting values directly, ecological shifts can also induce behavior change, which subsequently leads to value change, as occurred in a Maya village in Chiapas, Mexico (Greenfield 2004).

Subsistence ecologies are characterized by small villages, short life expectancies, low material resources, collectivism, and basic survival activities; people produce their own food, shelter, and clothing. Most relevant to our argument, members of subsistence ecologies prioritize practical intelligence over abstract intelligence and measure social intelligence by how well an individual can negotiate the social world to benefit the collective (family and community) rather than the individual (Greenfield 2019). Early in human history, these two forms of intelligence were successful adaptations for living in small-scale ecologies with high mortality rates and scarce resources. 

In commercial ecologies (a product of cultural evolution) most people live in large-scale urban environments; people have substantially longer life expectancies, access to more material resources, and purchase rather than produce food, shelter, and clothing. Most relevant to our argument, people living in a commercial environment prioritize abstract intelligence over practical intelligence; they measure social intelligence by how well an individual can negotiate the social world to benefit the individual rather than the collective (Greenfield 2019). These forms of intelligence are not mutually exclusive contrasts but are relative prioritizations of intelligence components.

Contrary to the conventional construct of intelligence as measured by IQ, we are taking intelligence as a multi-faceted construct consisting of an amalgamation of different sub-constructs that include ability, knowledge, character, wisdom, values, and skills. Our concept of intelligence also subsumes underlying motives or aims which employ these subconstructs.

## 3. The Pandemic Creates a Shift in Valued Forms of Intelligence

Our research has shown that, as COVID-19 increased mortality rates in the United States, made resources scarcer, and greatly reduced the scale of people’s social world, values and behavior shifted toward the values and behavior characteristic of subsistence ecologies (Evers et al. 2021; Greenfield et al. 2021), albeit in a technologically enhanced environment (Brown and Greenfield 2021). Since the pandemic made the environment radically more dangerous and people’s social world much smaller in a very short period of time, it served as a powerful natural experiment.

High mortality rates and small social units are characteristics of a subsistence village ecology. During the coronavirus pandemic, both of these environmental features increased greatly in a sudden fashion. Under stay-at-home orders, people were interacting with a smaller number of other people. At the same time, many were experiencing increased danger from COVID-19. Conditions were moving toward those found in subsistence ecologies.

Our theory predicted (and we found) that greater survival concerns (e.g., thinking about one’s own mortality) and more days spent observing stay-at-home rules would lead to increased subsistence activities and values, more collectivism, greater family interdependence, and parents socializing children to contribute to family maintenance. We tested the theory in the United States with both a large-scale survey and a massive analysis of internet behavior (Evers et al. 2021; Greenfield et al. 2021).

Most relevant to forms of intelligence, subsistence activities (e.g., growing food) are a manifestation of practical intelligence; the frequency of these activities rose during the pandemic both online and in the real world. Shifts in values and new directions in children’s socialization supported the development of practical intelligence. During the pandemic, subsistence values (e.g., conserving resources) increased and parents expected children to contribute more to household subsistence with practical skills (e.g., helping prepare family meals). This shift in parent expectations not only developed children’s practical intelligence; contributing to family subsistence may have also developed their *collectively oriented social intelligence.* Increases during the pandemic of collectivistic values (sacrificing, sharing, helping, and giving), as well as increased family interdependence (participation in family activities, mutual help to family members) were a manifestation in both values and behavior of the increased importance of collectively oriented social intelligence.

These findings represent a shift in American psychology and behavior toward that typical of the small-scale subsistence villages, prevalent at an earlier point in human history. Since we found “parallel adaptations occurring in only a few weeks during stay-at-home and the pandemic, we suggest that the human species is geared for the same adaptations when these conditions reappear” (Greenfield et al. 2021). However, societies where there was, even before the pandemic, greater respect for authority, a characteristic associated with subsistence ecologies, had an easier time responding to the pandemic with behavioral restrictions than societies, such as the United States, in which the value of individual freedom was greater (Gelfand 2020).

## 4. The History of Practical Intelligence in the Field of Psychology

The concept of practical intelligence emerged in the 1940s with the invention of situational judgment tests to assess managerial potential and came to the forefront with Robert Sternberg’s triarchic theory of intelligence and subsequent theory of successful intelligence (McDaniel and Whetzel 2005; Sternberg 1985, 1988, 1997). Practical intelligence has been characterized as “street smarts”, “common sense”, or the “cognitive underpinning of everyday function” (Hedlund 2020, p. 737; Yalon-Chamovitz and Greenspan 2005, p. 220; Nunes et al. 1993). More generally, practical intelligence refers to the ability to solve the problems individuals encounter in everyday life (Hedlund 2020; Sternberg and Grigorenko 2000). It requires employing solutions to problems that involve the actual doing of something and can be contrasted to abstract intelligence, employing solutions that concern theory and ideas.

Practical intelligence is a form of intelligence that evolved to facilitate the functioning and survival of subsistence communities (Greenfield 2019). Subsistence activities require the development of practical intelligence. However, practical intelligence is usually not prioritized in our technological environment, where the emphasis is on abstract intelligence. However, as noted earlier, during the pandemic, there was a significant rise in the exercise of practical intelligence (Evers et al. 2021; Greenfield et al. 2021). This shift was stimulated by increased mortality salience brought about by the all-too-real mortality threat of the pandemic and the narrowing of the social world to household and immediate neighbors due to stay-at-home orders (Greenfield et al. 2021). Activities that increased during the pandemic, such as baking bread or home repair (Evers et al. 2021; Greenfield et al. 2021) require practical intelligence, adapted to real-world contexts, as Alexander Luria (1976) pointed out decades ago.

The different intelligences are suited to the perceived urgency with which a problem needs to be solved. Practical intelligence is better suited to high levels of immediacy, and abstract intelligence is better suited to lower levels of immediacy. For this reason, in times of danger when humans face an immediate threat, they employ more practical intelligence than abstract intelligence and vice versa under safer conditions. This ability to shift the focus of intelligence in order to adapt to different conditions is a basic secret to the evolutionary success of human beings.

## 5. Types of Social Intelligence in the Field of Psychology

The modern concept of social intelligence has its origin in E.L. Thorndike’s division of intelligence into three different abilities: the ability to comprehend and manipulate ideas (abstract intelligence), concrete objects (mechanical intelligence), and people (social intelligence) (Thorndike and Stein 1937). Thorndike wrote: “By social intelligence is meant the ability to understand and manage men and women, boys and girls—to act wisely in human relations.” Since Thorndike’s classical formulation of social intelligence, it has been measured and defined in many ways, and two principal perspectives emerged (Kihlstrom and Cantor 2000). The first perspective was social intelligence as an ability, embodied by Thorndike’s definition above and subsequently espoused in different notable iterations by Guilford (1967), Gardner (1983), and Goleman (1995). Later, Kihlstrom and Cantor offered a second perspective, “The Knowledge View of Social Intelligence”, where social intelligence refers to an “individual’s fund of knowledge about the social world”, mediated by the individual’s general cognitive processes (Kihlstrom and Cantor 2000, p. 573; 1989; Cantor and Kihlstrom 1987, 1989).

However, these definitions assess small differences between individuals in today’s developed Western societies. This measurement orientation is evident in the individualistic lens through which social intelligence is defined both by Thorndike and Kihlstrom/Cantor: intelligence as an ability measures how well an individual can manipulate others for their aims. Intelligence as knowledge differs only slightly since it says that social intelligence is an individual’s store of specific knowledge about the social world transformed into behavior by general cognitive ability. Whether someone has social intelligence, defined as an ability or a fund of knowledge, their intelligence is measured by how well they can manipulate others for their own aims as an individual. This measure is most relevant for current Western psychology since the status quo of Western thinking and, as a result, psychology as a discipline, is to use the individual as the basic unit of society.

However, in this essay, our interest is in fundamental deviations from the status quo of Western thinking, shifts from the psychology and behavior of modern ecologies toward the psychology and behavior of the subsistence ecologies that defined an earlier era of human history and exist in pockets of today’s world. A fundamental difference between subsistence ecologies and ours is that, in the former, the welfare of the community supersedes that of the individual.

Outside the Western definitions of social intelligence as a measure of ability or a fund of knowledge, an entirely different conception has arisen through studying how subsistence ecologies think about what we would refer to as “social intelligence.” Mundy-Castle’s definition of social intelligence originated in his long-term experience with African cultures. He defined social intelligence to include character, wisdom, and collectivistic values, and emphasized that social intelligence incorporated technical skills insofar as they contributed to the community (Mundy-Castle 1974). Similarly, Dasen, studying a Baoulé village in Ivory Coast, emphasized that the Baoulé concept of intelligence, *n*’*glouèlê,* integrates cognitive and social skills, as do many other African concepts of intelligence (Dasen 2011). Indeed, the most central (in the sense of agreed upon) attribute for intelligent children listed by Baoulé farmers was “readiness to carry out tasks in the service of the family and the community”, a social quality (Dasen 1984, p. 426).

Lest one think that collectively oriented social intelligence is not suited to measurement, we note that a Pakistani team psychometrized a closely related concept of social intelligence, developing and validating a social intelligence scale in a sample of Pakistani university students (Habib et al. 2013). Their factor analysis revealed empathy, a key quality in collectively oriented social intelligence. as a principal component of social intelligence, a quality that had been declining in U.S. culture for decades (Konrath et al. 2011).

For our analysis, we distinguish the social intelligence of modern commercial ecologies from subsistence ecologies by the end goal. Whether we define social intelligence as a measure of ability or fund of knowledge, the social intelligence of modern commercial ecologies furthers the individual’s aims, whereas the social intelligence of subsistence ecologies furthers the collective’s aims. We connect social intelligence in subsistence ecologies to care for a broader community, not just oneself, and the willingness to make sacrifices for others. A rise in the social intelligence important in subsistence ecologies would increase someone’s motivation to care more about and make sacrifices for their broader community. Our thesis is that the rising value of this collectivistic form of social intelligence, as well as the increased value placed on practical intelligence, paved the way for large-scale social movements during the pandemic.

## 6. Shifts in Valued Forms of Intelligence Prime Americans for Social Movements

Our thesis is that the augmented value of practical intelligence and collectivistically oriented social intelligence resulted in a massive increase in the desire to solve problems that faced communities and to solve them using real-world action. This is the broad-strokes definition of a social movement. Our evidence is that the pandemic witnessed a number of social movements of unprecedented size and strength. In a relatively short period of eight months, three movements spanning ideology and form shook the country. These movements were all similar in that they were expressed through employing solutions that involved the actual doing of something (practical intelligence) and, in all three cases, their aim was to benefit a community (collectively oriented social intelligence). However, each movement served the goals of a different community, thus contributing evidence to the generality of the theory. When environmental danger increases, it shifts intelligences to prime communities to enact social movements, and the shift in valued intelligence implies a shift in values. However, social movements can still occur under other conditions.

Interestingly, despite these proclivities having developed in pre-technology communities, we see them play out in our technologically enhanced environment, a factor that strengthened all of the social movements. In fact, in our increasingly tech-enabled world, the internet is forming communities along unprecedented lines and creating ingroups based on various similarities, whereas, in the past, primarily geographic and ethnic similarities defined communities. Today, the internet can align like-minded folks and place them into information silos, which further entrench their allegiance to their community and strengthen their beliefs in their communities’ ideologies.

However, there was also increased interaction within small neighborhood units during the pandemic as geospatial research using cellphone data shows: the number of COVID-19 cases was correlated with increases in neighborhood isolation a week later. Additionally, places with larger populations, more public transportation use, and greater racial and ethnic segregation had larger increases in neighborhood isolation during 2020 (Marlow et al. 2021). Both the kind of communities that formed earlier in human history and those that have developed most recently grew stronger.

## 7. The George Floyd Protests

Incited by George Floyd’s death in police custody, the George Floyd Black Lives Matter protests became the “largest movement in U.S. History”, with an estimated 15 to 26 million adults taking to the streets to demonstrate (Buchanan et al. 2020). George Floyd was not the first black individual to be killed by the police or even to have their murder filmed, so why did his death in particular cause a cultural movement of unprecedented scale? Why did the largest social movement in U.S. history occur during the height of a deadly pandemic, which threatened Americans with the very real possibility of catching an illness that was ravaging the country around them?

Our theory makes the unlikely timing of this protest movement of unprecedented scale understandable. It posits that the dramatic increase in ecological danger increased collectively oriented social intelligence, priming people to want to solve issues that faced their community. The dramatic increases in the mindshare of “sacrifice”, “share”, “help”, and “give” observed in our online analyses (Evers et al. 2021) may have primed people to be more inclined to set aside their daily commitments, forget their hesitations to civil disobedience, and lessen the extent of their self-protective coronavirus measures to improve the welfare of the Black individuals that they viewed as members of their community.

Based on Reny and Newman (2021), it seems that the community driving the George Floyd protests was low-prejudice and politically-liberal Americans. Unlike prior Black Lives Matter protests, which were majority black and could be interpreted as individualistic responses to bettering one’s own welfare, almost 95% of the American counties that participated in the recent Black Lives Matter protest, were majority white, indicating an altruistic desire to help disenfranchised members of one’s community (Buchanan et al. 2020). Such a community did not exist in geographic space; it was formed virtually by means of the internet. This rise in altruistic action reflects an increased value placed on collectivistic components of social intelligence. At the same time, the rise in practical intelligence documented in our studies of the pandemic was expressed by practical action on the streets rather than more abstract virtual action online.

## 8. 2020 United States Presidential Election

Five months after the Black Lives Matter movement, an unprecedented social movement driven by two very different communities occurred. The 2020 United States presidential election on 3 November 2020, witnessed the highest voter turnout by percentage since 1900, with this voting spike occurring across both Democratic-leaning and Republic-leaning demographics (Park 2020; Frey 2021). The unusually high turnout by both Democrats and Republicans reflects the divided political landscape of America. As predicted by our theory, members of both parties felt a significantly increased desire to solve the problems facing their respective community, that is, members of their political party, and expressed that desire through the real-world solution of voting.

## 9. 2021 United States Capitol Attack

Two months after the election, on 6 January 2021, the United States Capitol was violently attacked by a mob of Donald Trump supporters, who successfully disrupted the planned counting of electoral votes that would formalize Joe Biden’s victory (Luke 2021; Reeves et al. 2021). The attack resulted in American insurrectionists mounting the first mass breach of the U.S. Capitol since the War of 1812 (Dilanian and Collins 2021; Lakritz 2021). Hundreds of Donald Trump supporters felt a significantly increased desire to solve problems facing their community; in this case the community was right-wing extremists, again very much a product of an internet information silo. They integrated this manifestation of social intelligence with the practical intelligence necessary to organize an attack on the Capitol to disrupt the counting of the electoral votes.

## 10. Conclusions

### 10.1. Implications for Social Change

When we initially found that residents of the United States valued practical intelligence and collectively oriented social intelligence significantly more during the pandemic, our interpretation was positive (Evers et al. 2021; Greenfield et al. 2021). We predicted that these shifts in valued intelligence would optimize the creation of a more cohesive and empathetic society, thus reversing the documented historical decline in empathy (Konrath et al. 2011) and communitarian activity (Putnam 2000). At the simplest level, we thought people would care more about other people and employ practical methods to realize their desired outcomes. At that point in the pandemic, the Black Lives Matter movement was the only social movement of unprecedented size and strength that had occurred, so, being low-prejudice, liberal Americans ourselves, we concluded that collectivistic goals combined with real-world action lend themselves particularly well to social progress. When the other two unprecedented movements occurred, it threw into question our idea of linear social progress. Today’s society is much larger than the social units of early human history. For most of cultural history, the social unit of reference was a small village in which everyone knew each other. However, the United States has a population of over 300 million (United States Census Bureau 2021). Our prediction that the shifts in valued intelligences would create linear social progress was wrong since social progress is subjective, and the functional collective unit is not the United States but subgroups within the country. People identify with their communities and, as the world gets more dangerous and protective responses kick in, those ingroups become even more cohesive. It seems clear that the shifts in valued intelligence created by increased mortality rate, resource scarcity, and a narrowing of the social world do not lead to any particular direction of social change but to stronger and larger social movements that have more to do with a specific community’s desired direction of social change than to the country as a whole. Hence, the result can and has been social movements going in opposite directions. In a complex society, collectively solving one community’s social problems may easily be perceived to create greater social problems for a different community.

### 10.2. How Lasting Are These Pandemic-Induced Intelligence Shifts?

As observed by Evers et al. (2021) and Greenfield et al. (2021), the pandemic caused humans to adapt very quickly. America witnessed a massive shift in psychology and behavior toward an earlier time of human history within a couple of months. Therefore, humans will likely adapt similarly quickly in the opposite direction when the conditions reverse.

### 10.3. The Adaptiveness of Shifting Intelligence

Intelligence is traditionally conceptualized as a general factor in a psychometrically-based hierarchical intelligence model and measured by standardized tests such as the IQ test (Sternberg 2019). Early psychometricians designed the IQ test to predict real-world performance, and the tests were considered valuable to the extent that they predicted that performance (Sternberg 2021). However, a false turnaround has occurred over time where the IQ or score on a similar standardized test of intelligence has become more important than whatever the tests predict. While almost all definitions of intelligence agree on one thing, that intelligence involves the ability to adapt to the environment, the current understanding of “intelligence” refers to a construct that is, at best, vaguely related to intelligence as adaptation (Sternberg 2019). Sternberg rejects the current understanding of intelligence as measured by IQ and similar measures and instead believes that the measure of intelligence should be its adaptiveness in an evolutionary sense (Sternberg 2019, 2021). Human psychological processes and behavior that are “adaptively intelligent” further the biological interests of survival. Sternberg defines adaptive intelligence in the context of success at broad adaptation. Broad adaptation includes narrow adaptation, a “process by which an animal or plant species becomes fitted to its environment; it is the result of natural selection’s acting upon heritable variation” (Gittleman 2018) along with “changing the environment to fit oneself (shaping the environment) and finding or creating new environments as needed (selecting environments)” (Sternberg 2019).

By this definition, would the observed shifts in intelligence be considered “adaptively intelligent?” Yes, but in a slightly different fashion than Sternberg’s model of adaptive intelligence. First, it appears that the shifts in intelligence observed during the pandemic occurred with the community as the basic social unit and furthered the aims of the community, which may or may not have aligned with the aims of the entire species. However, it is unnatural for humans to think on such a large level as optimizing the survival of the whole human species when we have been hard-wired through evolutionary history to operate on the level of a small village. For this reason, it seems people have a hard time making progress against the existential threats facing humanity, namely climate change, nuclear weapons, and pollution, which Sternberg argues would be some of the best measures for adaptive intelligence (Sternberg 2019). We would say that for better or for worse, evolution did not condition humans for those sorts of threats. They are too big and too distant. Instead, it seems that humans are much better suited to be adaptively intelligent on the level of their community or ingroup. We would posit that intelligence is a community, not an individual or species adaptation.

It is intuitively adaptive that when the world gets more dangerous, the rising value of collectively oriented social intelligence increases motivation to care more about and further the aims of one’s tribe, and the rising value of practical intelligence increases the motivation to solve the pressing issues facing their tribe using practical solutions. While this banding together as a community and solving the community’s pressing issues is generally a successful evolutionary strategy, it requires that the community solve the correct issues with the correct solutions. For these shifts in intelligence to be adaptively intelligent, they would have to focus on solving survival problems, which would mean that the community would have to care enough about the survival problems and address them with a correct solution. While, on average, humans have been able to do this successfully, that does not mean that the evolutionarily fit decision is always evident.

For example, one might say that refusing to wear masks caused thousands or hundreds of thousands of deaths. However, this is a matter about which different communities, split down partisan, gender, and racial lines, hold differing ideologies (Brenan 2020). One’s opinion on this issue says more about the community they belong to than the objective adaptivity of mask-wearing since both the communities that wear masks and those that refuse to can rationalize their behavior using the language of survival. Mask wearers can point to research showing that masks effectively decrease the spread of air-borne COVID-19 particles (Union-Bulletin Editorial Board 2020). In contrast, those who refuse to wear masks can point to research showing that wearing masks can lead to a dangerous false sense of security, cause other health risks, worsen the burden of COVID-19 on an individual, and even increase the spread of COVID-19 through inappropriate mask use (Lazzarino 2020).

Humans can never truly know the actions to execute that will best ensure the survival of their community. Therefore, they act on ideologies since, by definition, an ideology is the system of ideas held by a community. We have examples to show that when people come under threat, they shift these specific intelligences, resulting in furthering the collective’s aims through practical measures. However, it seems that the aims of the collective that this adaptation furthers have a higher correlation with the centrality to the community’s ideology than the objective adaptiveness of these aims as defined by survival. That said, one can imagine that pressing survival threats in forms that evolution conditioned humans for (not the massive, distant threats of climate change, nuclear weapons, and pollution) would become central to a community’s ideology and be solved. Therefore, it appears that humans use a heuristic to reason by proxy that the most adaptive path is to execute their community’s ideology. Does this mean that ideology trumps intelligence? No. When the world gets more dangerous, intelligence becomes the vehicle for enacting and furthering ideology. Why might this be evolutionarily fit? Since in dangerous times, individuals banding together as a collective to enact solutions in line with their community’s ideology is a safer and faster bet for problem solving than if the individuals were to innovate unique solutions.

### 10.4. Implications for Cultural Evolution

Different intelligences are adapted to different ecologies. For example, in pre-COVID times, abstract intelligence was valued over practical intelligence and *individually oriented social intelligence* was valued over collectively oriented social intelligence in the United States and other commercial ecologies (Mundy-Castle 1974; Greenfield 2019). These forms of intelligence are perfect for making technological and scientific progress. As evinced by rising economic inequality and other social problems, this progress came at the cost of having an increasingly less empathetic and community-minded society (Konrath et al. 2011; Putnam 2000). As valued intelligences shift, based on changing ecological conditions, there will always be a trade-off between the psychological conditions that best push humanity into the future through technological and scientific advances and the conditions that strengthen social bonds and lead to solving social problems.

This dynamic interplay between valuing community welfare and valuing technological progress is, in our view, a permanent part of human history. We hypothesize an evolved tendency in social and cultural evolution to favor groups that strengthen their social units in times of danger and instability but push forward with advances in technology and abstract thinking in times of safety and stability. If corroborated by continuing research, the balance between these opposing forces would even appear to be responsible for much of humankind’s evolutionary success.
